# Efficacy of an optimal ovarian cancer screening: a best-case scenario study based on real-world data

**DOI:** 10.1007/s00404-021-06117-4

**Published:** 2021-06-14

**Authors:** Lena Steinkasserer, Delmarko Irmgard, Tatjana Weiss, Walter Dirschlmayer, Michael Mossig, Alain G. Zeimet, Christian Marth

**Affiliations:** 1 Department of Obstetrics and Gynecology, Medical University Innsbruck, Anichstrasse 35, 6020 Innsbruck, Austria; 2 Department of Clinical Epidemiology, Tyrolean Federal Institute for Integrated Care, Tirol Kliniken GmbH, Innsbruck, Austria; 3 Department of Obstetrics and Gynecology, Hospital Barmherzige Schwestern, Linz, Austria; 4 Department of Obstetrics and Gynecology, Hospital Barmherzige Schwestern Ried, Vienna, Austria; 5 Department of Obstetrics and Gynecology, Hospital Hietzing, Vienna, Austria

**Keywords:** Ovarian carcinoma, Cancer Screening, Survival rate, Stage-shift

## Abstract

**Purpose:**

To date, ovarian cancer screening in asymptomatic women has not shown a mortality benefit. The aim of this simulation study was to outline the impact of different histological subtypes on a potential stage-shift, achieved by screening.

**Methods:**

Real-world data were derived in the period of 2000–2017 from the Klinischen Tumorregister Austria. We estimated five-year overall survival (OS) of patients with ovarian cancer regarding different histological subtypes and FIGO stages. A theoretical model was generated predicting the trend of OS mediated by an eventual down-shifting of ovarian cancer from FIGO stage III/IV to FIGO stage I/II by screening, considering the influence of different histological subtypes.

**Results:**

3458 ovarian cancer patients were subdivided according to histological subtypes and FIGO classification. Major difference in distribution of histological types was found between FIGO stage I/II and III/IV. A theoretical down-shift of tumors from high to low FIGO stages based on our registry calculations showed that the five-year OS would increase from 50% to nearly 80% by perfect screening.

**Conclusion:**

In our simulation study, we showed that down-shifting ovarian cancers by successful screening might increase OS by 30 percentage point. Our results underscore the importance to recognize ovarian cancer as a heterogenous disease with distinct epidemiologic, molecular and clinical features. The individual characteristic of each histotype is of utmost impact on the definition of screening aims and may influence early detection and stage-shift. Efficacy of screening is mainly dependent on detection of high-risk cancer types and not the slow growing low-grade types.

## Introduction

Screening as an option to detect cancer early to improve OS is a desirable medical tool. Although screening represents a laudable strategy to detect a potential cancer in an early stage and to ensure a subsequent therapy, it also contains the risk of over-diagnosis [1–5]. To guarantee that screening of asymptomatic patients represents a benefit for the target population, it needs to be characterized by some factors: a well-defined target population, abilities for diagnosis and treatment, detectable latent or early symptomatic stage, suitable test or examination and many others. The primary aim is to spot and remove the precancerous lesions to prevent a premalignant lesion from progressing into an invasive cancer [4–7]. Cervical cancer screening and colorectal cancer screening fulfill most of the conditions and represent therefore examples of public health prevention programs with significant success and are two of the most widely used approaches to screen cancers [6, 8–12]. However, ovarian carcinoma is difficult to diagnose in early stages. It is major challenge to detect precursor lesion such as serous tubal intraepithelial carcinomas (STIC), the cancer has a low prevalence with a lifetime risk of approximately 1.2–2%, a short transit time, the origin differs among the different histological subtypes and the high false-positive tests lead to a high morbidity due to unnecessary surgical interventions [6]. The majority of ovarian cancers are diagnosed in an advanced stage with the consequence, that the survival rate is low. Therefore, early detection of ovarian cancer using screening is an aim which has been strived for in the last several decades. There are two large prospective trials which investigated the mortality benefit of screening: the “UK Collaborative Trial of Ovarian Cancer Screening (UKCTOCS)” and the “The Prostate, Lung, Colorectal and Ovarian Screening Trial (PLCO)”. So far, no trial was able to show any significant mortality benefit for women at average risk using screening [13, 14]. Accordingly, the US Preventive Services Task Force (USPSTF) has reported that major trials of promising ovarian cancer screening tools did not show a mortality reduction among healthy average risk women. If anything, they reported considerable harms associated with screening [15]. Recently, the understanding of ovarian cancer as a heterogeneous disease with distinct morphology, origin and molecular pathogenesis has underscored the importance to strictly distinguish between the various histological subtypes. This knowledge may have an impact on the efficacy of ovarian cancer screening [16–19].

The primary aim of this best-case scenario study was to elucidate the effect of a potential stage-shift on OS in ovarian cancer screening, when the distinct impact of the various histological subtypes is taken into consideration.

## Methods

### Patient cohort

Cases were identified retrospectively by computer search in the database of the Klinisches Tumorregister Austria, a cancer registry organized by the Austrian Association for Gynecologic Oncology (AGO). The outcome of the procedure was documented prospectively in patients’ record forms. Histology was taken from the local pathology and no central review has been performed.

### Clinical and demographic data

All clinical data were evaluated from the database. Clinical data included the FIGO stage of the diagnosed carcinoma as well as its histological subtype. Based on the survival rates of the individual FIGO stages (FIGO stage I/II and FIGO stage III/IV), the overall survival (OS) across all stages for our real-world data was calculated**.**

### Histological subtypes

Carcinoma subtypes were classified according to their different clinical and biological behavior into high-grade serous carcinomas (HGSOC), high-grade endometrioid carcinomas (HGEOC), low-grade serous carcinomas (LGSOC), low-grade endometrioid carcinomas (LGEOC), mucinous carcinomas (MOC), clear cell carcinomas (CCOC) according to the recommendation of the fifth ovarian consensus conference [20|. Furthermore, the FIGO classification 2014 was used to subdivide the different histological subtypes into two groups: FIGO I/II and FIGO III/IV [21].

### Statistics

Potential differences in five-year OS by histological subtypes (HGSOC, HGEOC, LGSOC, LGEOC, MOC, CCOC) and FIGO stages (FIGO stage I/II and FIGO stage III/IV) were analyzed using the product limit method of Kaplan–Meier and the log-rank test. Kaplan–Meier curves were generated using Stata V15/IC (StataCorp LLC; 4905 Lakeway Drive; College Station, TX 77,845; USA).* χ *^2^ test was used to determine the differences in FIGO stage and histology. In this retrospective study, p values should be viewed with caution due to the fact that in large samples even minuscule effects can become statistically significant [22].

## Results

**Fig. 1:**
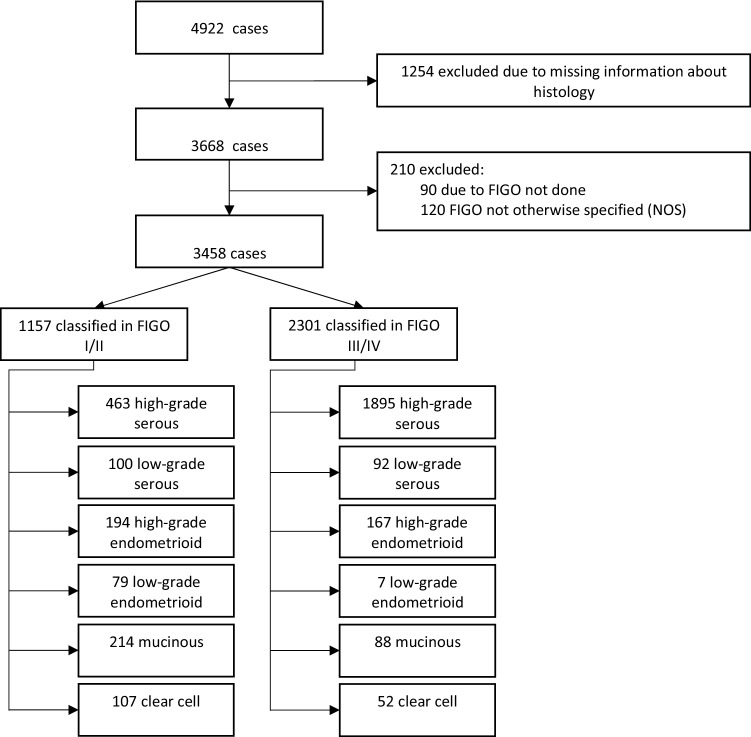
Consort diagram

**Fig. 2:**
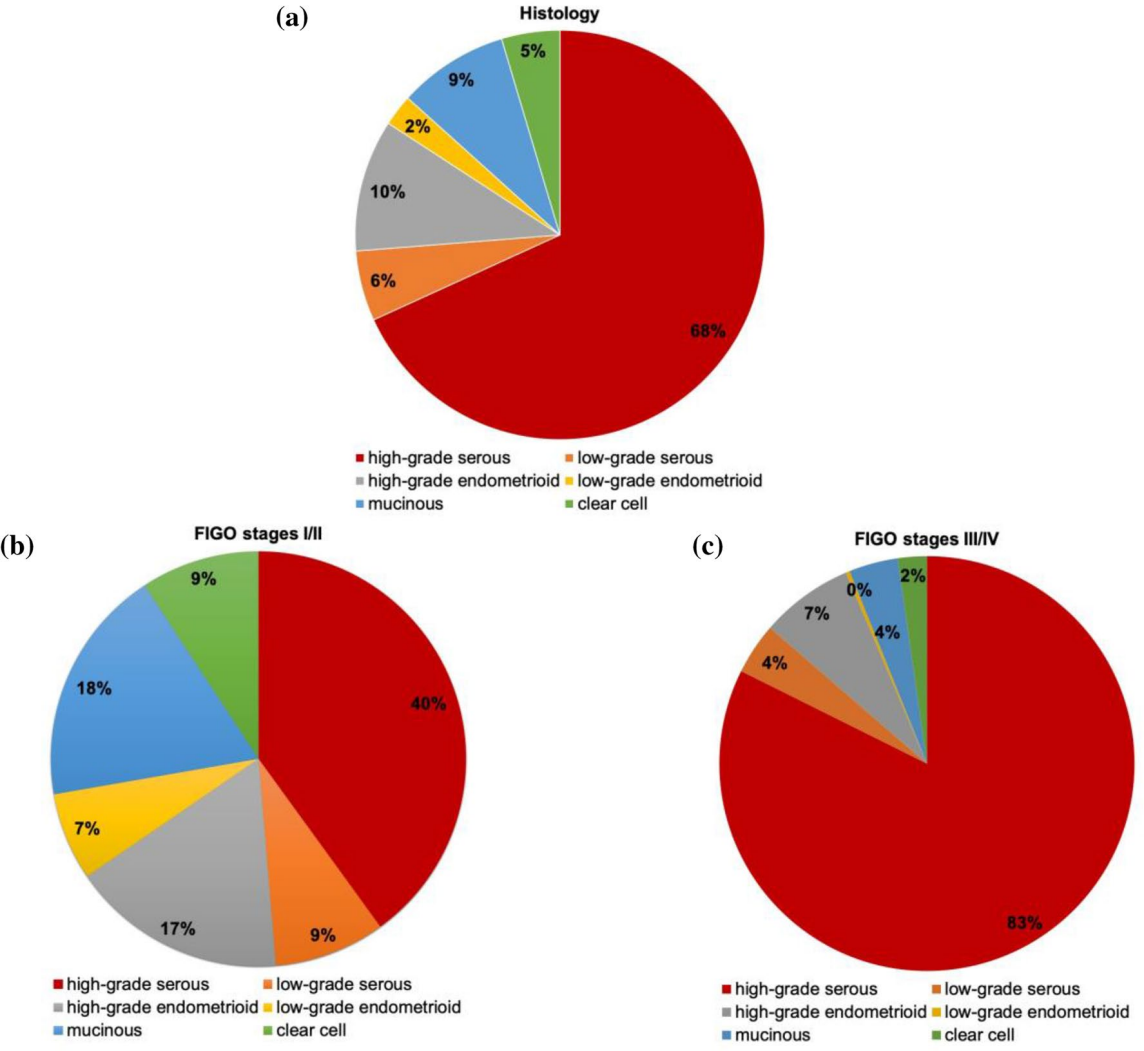
**a** FIGO stage distribution among study population **b** Distribution of histological subtypes of ovarian cancers in FIGO stage I/II; *n* = 1157; high-grade serous = 463; low-grade serous = 100; high-grade endometrioid = 194; low-grade endometrioid = 79; mucinous = 214; clear cell = 107 **c** Distribution of histological subtypes of ovarian cancers in FIGO stage III/IV; n = 2301; high-grade serous = 1895; low-grade serous = 92; high-grade endometrioid = 167; low-grade endometrioid = 7; mucinous = 88; clear cell = 52

A total of 4922 ovarian cancer patients diagnosed and treated in different Austrian clinical centers (Table. 1) between 2000 and 2017 were included for this retrospective study. This represents 37% of all ovarian cancer patients diagnosed in Austria during this time period. 1464 (30%) cases were excluded due to missing information about the exact histology or the lack of information on FIGO stage. The final study population comprised a total of 3458 cases (70%). The cases were classified with respect to the FIGO stage, then pooled in either FIGO stage I/II or FIGO stage III/IV (Fig. 1) and further subdivided for their respective histological subtype (Fig. 2a). Combined distribution according to stage and histological subtype is shown in Fig. 2b, c. It is interesting to mention, that the distribution of the histological types was highly different in both FIGO stage groups (chi-square 702,27, df = 5, *p* < 0001). Proportion of HGSOC was 40% and 83% in FIGO stage I/II and III/IV, respectively.

**Table 1:** Clinical centers

City	Department
Innsbruck	Medical University
Wien	Hospital Wilhelminens
	Hospital Kreuzschwestern
	Hospital Barmherzige Brüder
	Hospital Hietzing
	Hospital Rudolfsstiftung
	Hospital Hanusch-KH
	Hospital SMZ-Ost
	Hospital Kaiser-Franz-Josef
	Hospital Göttlicher Heiland
	Hospital St. Josefs
Linz	Hospital Barmherzige Schwestern
	Hospital Elisabethinen
	State Women's and Children's Clinic
	Hospital
	Hospital Barmherzige Brüder
Salzburg	Hospital St. Johannsspital
	Hospital Barmherzige Brüder
Graz	Medical University
	Hospital Barmherzige Brüder
Kittsee	Hospital KRAGES
Oberwart	Hospital KRAGES
Kufstein	District Hospital
Ried/Innkreis	Hosptial Barmherzigen Schwestern
St. Pölten	University Hospital
Wiener Neustadt	State Hospital
Krems	University Hospital
Steyr	Hospital
Horn	State Hospital
Amstetten	State Hospital
Hall in Tirol	State Hospital
Wolfsberg	State Hospital
Mistelbach	State Hospital
Vöcklabruck	State Hospital
Freistadt	Hospital
Feldkirch	State Hospital
Pongau	Hospital Kardinal Schwarzenberg
Eisenstadt	Hospital Barmherzige Brüder
Braunau	Hospital Sankt Josef
Mödling	State Hospital
Bad Ischl	Hospital Salzkammergut
Rohrbach	State Hospital
Dornbirn	City Hospital
St. Veit	Hospital Barmherzige Brüder
Lienz	District Hospital
Scheibbs	State Hospital
Zwettl	State Hospital
Waidhofen/Thaya	State Hospital
Hallein	State Hospital
Baden	State Hospital
Lilienfeld	State Hospital
Hollabrunn	State Hospital
Spittal a. d. Drau	Hospital
Korneuburg	State Hospital
Judenburg-Knittelfeld	State Hospital
Neunkirchen	State Hospital
Gmunden	State Hospital
Rottenmann	State Hospital
Melk	State Hospital
Tamsweg	State Hospital
Tulln	University Hospital
Schärding	Hospital
Villach	Private Clinic
Klosterneuburg	State Hospital
Bruck an der Mur	State Hospital
Feldbach	State Hospital
Leoben	State Hospital
Hartberg	State Hospital
Oberndorf	State Hospital
Bregenz	State Hospital
Kirchdorf an der Krems	Hospital
Waidhofen/Ybbs	State Hospital
Deutschlandsberg	State Hospital

**Fig. 3:**
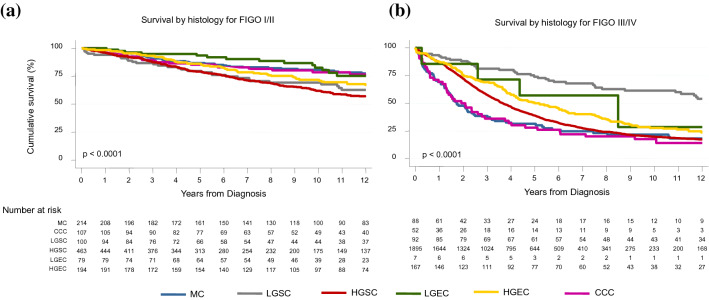
Kaplan–Meier curve of ovarian cancer survival by FIGO stage and histology. **a** comparison of survival between ovarian carcinomas in different histological subtypes in FIGO stage I/II **b** comparison of survival between ovarian carcinomas in different histological subtypes in FIGO stage III/IV

As shown in the Kaplan–Meier curves, OS was significantly different when comparing the various histological subtypes in FIGO stage I/II (p < 0.0001) (Five-year OS: MOC = 85%, CCOC = 83, LGEOC = 92%, LGSOC = 76%, HGEOC = 83%, HGSOC = 75%) (Fig. 3a).

The difference in OS was also significant when comparing the different histological subtypes in advanced FIGO stage (III/IV) (p < 0.0001) (Five-year OS: MOC = 26%, CCOC = 26%, LGEOC = 57%, LGSOC = 75%, HGEOC = 50%, HGSOC = 43%) (Fig. 3b).

In a post hoc adjustment, low-grade (LGOC) and high-grade carcinomas (HGOC) of the main two histological subtypes (serous and endometrioid) were combined to form two large groups and both groups were further sub-classified with regard to their FIGO stages (Table 2).

**Table 2:** High-grade group and low-grade group

	Total	FIGO I/II	FIGO III/IV
High-grade	2719; (79%*)	657 (24.2%)	2062 (75.8%)
Low-grade	278; (8%*)	179 (64.4%)	99 (35.6%)

*based on the study population

**Fig. 4:**
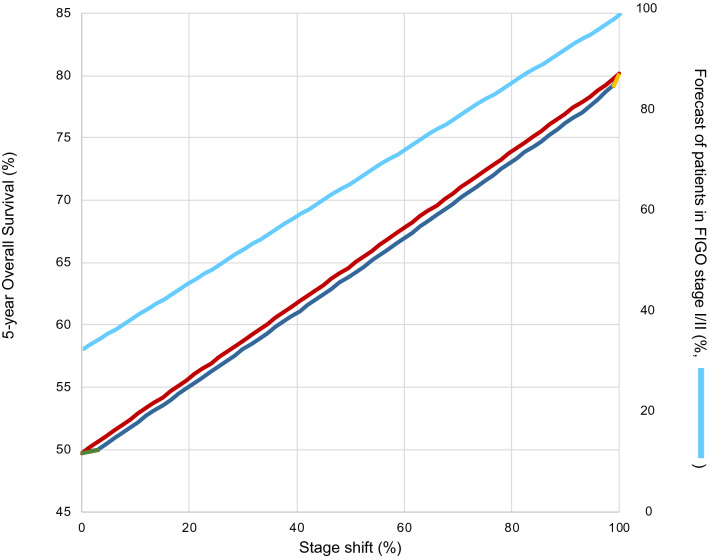
Effect of potential stage-shift from FIGO stage III/IV to FIGO stage I/II on five-year OS and forecast of patients on FIGO stage I/II. Red graph: stage-shift for ovarian cancer when no histological distribution was taken into consideration. Three-colored-curve: stage-shift when considering the different tendencies of different histological subtypes to be detected by screening and thus different effects on OS (LGOC: green line; HGOC/CCOC: blue line; MOC: yellow line)

To test for an improvement of five-year OS in women successfully screened for ovarian carcinoma, a diagram was elaborated simulating the effect of a potential stage-shift. For this purpose, five-year OS (x-axis) is plotted against the potential percentage of stage-shift (y-axis) (Fig. 4). In our cohort, five-year OS for ovarian cancer diagnosed in FIGO stage I/II was 80% and five-year OS in FIGO stage III/IV was 34%. The initial value (50%) represents the five-year OS rate across all stages based on our real-world data and corresponded to the current stage distribution of 33% ovarian carcinomas in FIGO stage I/II and 67% diagnosed in FIGO stage III/IV. Proportion of FIGO stage I/II was gradually increased and concurrently proportion of FIGO stage III/IV decreased. By including all histological subtypes together, a down-shift of 100% from FIGO stage III/IV towards FIGO stage I/II corresponded to an increase of 30 percentage point in OS. As an example, when 50% stage-shift would occur, the five-year OS would increase to 65% (red curve in Fig. 4).

We assume that probability for early detection of LGOC is much higher because those tumors take longer to proceed from an early to an advanced stage. This is also supported by the higher proportion of LGOC diagnosed in FIGO stage I/II. If screening-mediated stage-shift would affect mainly LGOC, the beneficial effect would be minor. The three-colored curve therefore initially showed a very flat slope when plotting five-year OS against stage-shift for low-grade carcinomas (green part in Fig. 4).

HGOC and CCOC are less likely to be detected early by screening due to their short transit time to progress from early to advanced stages. Nonetheless, these tumors showed a relatively good prognosis when found in an early stage but when diagnosed in an advanced stage they come along with a much poorer clinical outcome and OS. Thus, a successful screening stage-shift of these tumors would have the biggest impact on the improvement of the OS rate in ovarian cancer. This was depicted in the diagram with a much steeper slope (blue part in Fig. 4).

Due to the relatively low numbers diagnosed in FIGO III/IV**,** the impact of the MOC on the OS would be minor. Nevertheless, the early detection of this histological subgroup would have a moderate but relevant impact on the OS, because of the very poor prognosis in advanced stages (26%) as compared to the very favorable prognosis in early stages (83%). In total, the effect of early detection of MOC on the OS was shown in the last 2.5% of the curve (yellow part of the three-colored-curve in Fig. 4).

By consideration of the different impact of the distinct histological subtypes, the stage-shift of 100% from FIGO stage III/IV towards FIGO stage I/II corresponded to an increase of 30.1 percentage point in OS, leading from 49.7 to 79.8%.

## Discussion

Although screening can allow detection of ovarian cancer at an early stage, the used screening methods are not specific enough. Due to this and because of the high false-positive rate which caused consequential morbidity, screening examination in asymptomatic patients has not been recommended as a routine approach to date.

Theoretically, the early detection by screening would represent a considerable advantage for affected patients due to a better prognosis. However, the major question is, whether this advantage of an early diagnosis is not mainly due to the detection of exactly those cancers, which exhibit an a priori favorable, less aggressive behavior associated with better prognosis and is missing those which spread rapidly and are qualified as highly malignant. Herein, a theoretical down-shift of all the tumors irrespective of their histological subtype from FIGO stages III/IV to FIGO stages I/II would result in a five-year survival rate of approximately 80% (i.e., empiric five-year OS rate in FIGO I/II stages from our registry). These crude effects from stage-shift are caused by the respective incidence of the various histological subtypes and their real-world distribution in between the FIGO stages.

One strength of this study was that the consideration of the histological subtype was complemented by integrating the grade of malignancy. Into agreement with the World Health Organization (WHO) classification in the guidelines for female reproductive tumors in 2014, we classified the endometrioid cancer further into LGEOC and HGEOC [20, 21]. This gave us the possibility to investigate even more precisely the features of each histological subtype on the OS. The analyses were limited by the relatively high number of excluded cases, due to missing information with respect to the exact histological diagnose or FIGO stage. Needless to say, that p values as here provided have to be interpreted with caution because as in large case numbers, even small differences may become significant [22]. Another limitation was the absence of central pathology review. Although reproducibility among different pathologists regarding the grade of malignancy is good, no standardization regarding the histological classification of the cancers based on homogenous rules, has been applied [23–27]. A recently published study by Peres et al. included 28,118 epithelial ovarian cancer cases and estimated Kaplan–Meier survival curves by histotype and disease stage [28]. In comparison, our data revealed a higher five-year OS rate for each histological subgroup. This was especially true for the five-year OS rate for advanced LGSOC with 75% five-year OS in our study and 54% in the Peres study. Whereas Peres et al. reported a significant difference in the five-year OS between early and advanced LGSOC (93% and 54%, respectively), our results did not show such a difference between FIGO stage I/II (76%) and FIGO stage III/IV (75%) for these tumors. Furthermore, in FIGO stage I/II, the report of Peres et al., displayed as expected, significant poorer OS in HGSOC (84%) than in LGSOC (93%). In our study, however, the LGSOC and HGSOC diagnosed in FIGO stage I/II showed similar OS rates (76% vs 75%, respectively). Regarding this finding, it is striking that the OS rate of 76% in LGSOC in our cohort is poor in comparison with the results of Peres et al. Due to the absence of a central pathology review and the lack of a more profound molecular characterization by immunohistochemistry, we cannot exclude that some of the HGSOC have been misclassified as LGSOC in our collective and would thus explain the discrepancy of our findings to those of Peres et al. Nonetheless, the herein examined real-world data are tempting us to speculate that five-year OS is more strongly linked to the “stage of disease” than to the “malignant phonotype” of the cancers. Therefore, our findings do not support the above-mentioned hypothetical doubts that screening-related stage-shift would not be translated adequately on mortality benefit due to a selective detection bias in favor of slow growing, less aggressive cancers.

The PLCO study did not report any significant reduction of mortality by screening women at average risk [29]. In a subsequent follow-up of the of PLCO study, the outcome of the previous study was improved, since distinction between type I and type II cancers has been performed. Interestingly, type I tumors showed an improvement in the survival rate, when diagnosed earlier and could also be detected by screening at a higher rate compared to type II tumors. [13]. This supports our suggestion that the different histologic subtypes have a distinct tendency to be detected by the currently available screening methods and stage-shift has therefore an individual impact on five-year OS. The UKCTOCS study group reported a not significant mortality reduction of 15% in the MMS group in the primary analysis leading from 40% five-year OS to 55% OS [14]. In our best-case model, achievement of a five-year OS of 55% would require a stage-shift of 21 percentage point. This means, that 47% of the ovarian cancers should be diagnosed in FIGO stage I/II and 53% in FIGO stage III/IV. However, a significant mortality reduction of up to 28% in a pre-specified secondary subgroup analysis was suggested in the UKCTOCS trail, when prevalent cases were excluded [14]. Such a 28% mortality reduction assumes a five-year OS of 68% and would imply a stage-shift of 65 percentage point in our model, with 76% ovarian carcinoma diagnosed in FIGO stages I/II and 24% in FIGO stage III/IV. In the UKCTOCS, however, ovarian cancers were roughly defined as “*malignant neoplasms*” of the ovary, without differentiation between different histological entities, their resulting different biologic behavior and their different probabilities to be detected by screening [14].

In conclusion, we created a theoretical scenario, where we assumed every ovarian cancer can be early detected using not otherwise specified screening methods. In this set-up based on real-world data, we simulated the impact of a down-shift from FIGO stage III/IV to FIGO stage I/II on clinical outcome. Even though the stage-shift of every histologic subtype would increase the survival, early detection of HGOC and CCOC would have the biggest impact on OS benefit. Regarding screening endeavors in general, our data underscore the considerable influence of the “stage of ovarian cancer” on patients’ survival and highlights *“stage down-shifting”* as a proper tool to improve OS. Needless to say, that further research is required to explore the predictive power of screening methods especially regarding the different subtypes of ovarian cancer.

## Data Availability

Raw data were generated at Department of Clinical Epidemiology, Tyrolean Federal Institute for Integrated Care, Tirol Kliniken GmbH, Innsbruck, Austria. Derived data supporting the findings of this study are available from the corresponding author on request.

## Code availability

The code that supports the findings of this study is available from the corresponding authors upon reasonable requests.
